# Supplementary material for: Insights into the Multi-Azole Resistance Profile in Candida haemulonii Species Complex
Source: J Fungi (Basel). 2020 Oct 11;6(4):215. doi: 10.3390/jof6040215 (PMC7711680; doi:10.3390/jof6040215)
Supplement: Supplementary file 1 [file jof-06-00215-s001.pdf]

Journal of Fungi

MDPI

SUPPLEMENTARY DATA

Insights into the multi-azole resistance profile in *Candida haemulonii* species complex

Laura Nunes Silva [1], Lívia de Souza Ramos [1], Simone Santiago Carvalho Oliveira[1], Lucas Barros Magalhães[1], Eamim Daidrê Squizani[2], Lívia Kmetzsch[2], Marilene Henning Vainstein[2], Marta Helena Branquinha [1] and André Luis Souza dos Santos [1,3*]

[1] Laboratório de Estudos Avançados de Microrganismos Emergentes e Resistentes (LEAMER), Departamento de Microbiologia Geral, Instituto de Microbiologia Paulo de Góes (IMPG), Universidade Federal do Rio de Janeiro (UFRJ), Rio de Janeiro 21941-901, Brazil; lauransilva@gmail.com (LNS); liviaramos2@yahoo.com.br (LSR); simonesantiagorj@yahoo.com.br (SSCO); lbarrosmagalhaes@gmail.com (LBM); mbranquinha@micro.ufrj.br (MHB); andre@micro.ufrj.br (ALSS)

[2] Centro de Biotecnologia, Universidade Federal do Rio Grande do Sul (UFRGS), Porto Alegre, Rio Grande do Sul, Brazil; eamimsquizani@gmail.com; liviak@cbiot.ufrgs.br (LK); mhv@cbiot.ufrgs.br (MHV)

[3] Programa de Pós-Graduação em Bioquímica (PPGBq), Instituto de Química (IQ), Universidade Federal do Rio de Janeiro (UFRJ), Rio de Janeiro 21941-909, Brazil

* Correspondence: andre@micro.ufrj.br; Tel.: +55 21 3938-0366

Received: date; Accepted: date; Published: date

TABLE S1. Antifungal susceptibility profiles of the clinical isolates belonging to the *Candida haemulonii* species complex to different azole agents.

C. haemulonii **complex**	**MIC (mg/L)** [a]				
Isolates	**FLC**	**ITC**	**VRC**	**PSC**	**KTC**
C. haemulonii					
LIP*Ch*2	>64 (**R**)	1 (**R**)	0.5 (S)	8 (**R**)	4
LIP*Ch*3	>64 (**R**)	>16 (**R**)	16 (**R**)	>16 (**R**)	2
LIP*Ch*4	>64 (**R**)	>16 (**R**)	>16 (**R**)	8 (**R**)	2
LIP*Ch*7	>64 (**R**)	16 (**R**)	16 (**R**)	0.125 (**R**)	0.5
LIP*Ch*12	>64 (**R**)	16 (**R**)	16 (**R**)	0.5 (**R**)	2
C. duobushaemulonii					
LIP*Ch*1	>64 (**R**)	8 (**R**)	>16 (**R**)	0.03 (S)	1
LIP*Ch*6	64 (**R**)	0.5 (S-DD)	1 (S)	1 (**R**)	4
LIP*Ch*8	>64 (**R**)	8 (**R**)	>16 (**R**)	>16 (**R**)	>16
LIP*Ch*10	>64 (**R**)	0.25 (S-DD)	0.25 (S)	0.5 (**R**)	2
C. haemulonii **var.** ***vulnera***					
LIP*Ch*5	64 (**R**)	0.5 (S-DD)	0.5 (S)	0.03 (S)	0.125
LIP*Ch*9	64 (**R**)	8 (**R**)	16 (**R**)	0.25 (**R**)	0.5
LIP*Ch*11	>64 (**R**)	16 (**R**)	>16 (**R**)	0.03 (S)	1

[a] MIC, minimal inhibitory concentration; FLC, fluconazole; ITC, itraconazole, VRC, voriconazole; PSC, posaconazole; KTC, ketaconazole. R, resistant; S-DD, susceptible-dose dependent; S, susceptible. The clinical breakpoints defined for *Candida* spp. were used for the interpretation of MIC data as a follows: susceptible (S) ≤8 mg/L, susceptible-dose dependent (S-DD) 16-32 mg/L, resistant (R) ≥64 mg/L for FLC; S ≤0.125 mg/L, S-DD 0.25-0.5 mg/L, R ≥1 mg/L for ITC; S ≤1 mg/L, S-DD 2 mg/L, R ≥4 mg/L for VRC. For PSC, the susceptibility breakpoint was considered 0.06 mg/L, determined by EUCAST for *Candida* spp. No interpretive criteria for ketoconazole are available in the CLSI or EUCAST documents.

Table S2. **Antifungal susceptibility profiles of the non-*albicans* *Candida* species to different azole agents.**

***Candida* species**	**MIC (mg/L)** [a]				
	**FLC**	**ITC**	**VRC**	**PSC**	**KTC**
C. tropicalis (ATCC 750)	0.5 (S)	0.03 (S)	0.03 (S)	0.03 (S)	0.03
C. krusei (ATCC 6258)	>64 (**R**)	1 (**R**)	0.5 (S)	0.25 (S)	0.125
C. lusitaneae (ATCC 200950)	1 (S)	0.06 (S)	0.06 (S)	0.06 (S)	0.03

[a] MIC, minimal inhibitory concentration; FLC, fluconazole; ITC, itraconazole, VRC, voriconazole; PSC, posaconazole; KTC, ketaconazole. R, resistant; S-DD, susceptible-dose dependent; S, susceptible.

TABLE S3. **Homology search results comparing *Candida albicans* (SC5314) and *Candida haemulonii* (B11899) genes via BLAST.**

C. albicans (SC5314) genes	GenBank nucleotide accession number	*C. haemulonii* (B11899) genes	GenBank nucleotide accession number	Identity (%)	E value
CDR1	XM_718116.2	*ChCDR1*	XM_025483931.1	76.,61	3×10^{-137}
CDR2	XM_718076.2	*ChCDR2*	XM_025488660.1	74.58	8×10^{-81}
MDR1	XM_714072.2	*ChMDR1*	XM_025484012.1	73.84	3×10^{-20}
ERG11	XM_711668.2	*ChERG11*	XM_025486744.1	69.38	3×10^{-163}
